# Physical activity in adults with and without diabetes: from the ‘high-risk’ approach to the ‘population-based’ approach of prevention

**DOI:** 10.1186/1471-2458-13-1002

**Published:** 2013-10-24

**Authors:** Abla Mehio Sibai, Christy Costanian, Rania Tohme, Shafika Assaad, Nahla Hwalla

**Affiliations:** 1 Department of Epidemiology and Population Health, Faculty of Health Sciences, American University of Beirut (AUB), PO Box: 11-0236, Riad el Solh, Beirut 1107-2020, Beirut-Lebanon; 2 Affiliation at the time of the study: Department of Epidemiology and Population Health, Faculty of Health Sciences, American University of Beirut, Beirut, Lebanon; 3 Current affiliation: Centers for Disease Control and Prevention, Atlanta, GA 30333, USA; 4 Lebanese University, Faculty of Medical Sciences, Beirut, Lebanon; 5 Department of Nutrition and Food Science, Faculty of Agricultural and Food Sciences, American University of Beirut, Beirut, Lebanon; 6 Member of the Public Health and Nutrition (PHAN) Research Group at the American, University of Beirut, Beirut, Lebanon

**Keywords:** Type 2 diabetes mellitus, Lebanon, Physical activity

## Abstract

**Background:**

The prevalence rates of physical inactivity and diabetes in the MENA region are among the highest in the world. However, studies that focus on factors that influence the pattern of physical activity in the region remain very scarce. This study aimed to determine the prevalence and correlates of physical activity in the general population and among subjects with and without diabetes in Lebanon, a small middle-income country in the MENA region.

**Methods:**

We conducted a cross-sectional nation-wide study of 2,195 randomly selected adults aged 25 years and older. Participants completed a comprehensive questionnaire based on the WHO-STEPwise guidelines. Physical activity was assessed using the International Physical Activity Questionnaire. Type 2 diabetes risk factors examined were age ≥ 45 years, BMI ≥ 25 kg/m^2^, hypertension, hyperlipidemia, cardiovascular disease and family history of diabetes.

**Results:**

Close to 10% of adults with diabetes were physically active versus 23·4% without diabetes. Prevalence rates of physical activity declined consistently as the number of diabetes risk factors increased. Odds ratios for physical activity were lower among the educated (0.75, 95% CI= 0.57–0.98), those who owned at least one car (0.71, 95% CI= 0.57–0.88) and those who resided in the capital city (0.62, 95% CI 0.47–0.83). Health professionals gave ‘advice to exercise’ most to patients with or at highest risk for diabetes, and these were more likely to engage in physical activity than those without diabetes receiving the same advice, net of the effect of other covariates (OR=3.68 and 1.17, respectively).

**Conclusions:**

The inverse associations between physical activity and SES indicators suggest a negative influence of urbanization on activity levels of Lebanese adults. The missed opportunity for clinical primary preventive services for the majority non-diabetic population calls for population-based public health approaches that promote physical activity as a routine lifestyle in the general population.

## Background

The overall health benefits of physical activity have long been established. In 1953, Morris and colleagues showed that bus conductors in London experienced half the coronary heart disease mortality rates of their driver counterparts who spent their working days sitting behind the wheel [1]. Physical activity (PA) diminishes the risk of cardiovascular risk factors, and its role, notably in the prevention and management of type 2 diabetes, is well documented [2,3]. Today, lack of physical activity is the fourth leading risk factor for global mortality [4] and is one of the leading public health indicators [5]. Despite the known hazards of being sedentary, physical activity levels continue to fall worldwide [4]. In one article of the Lancet series dedicated to physical activity, Hallal and colleagues [6] note that roughly three out of ten adults aged 15 years and over are below the recommended physical activity threshold, and urge governments and populations to take physical inactivity as serious public health issue. The Middle East and North Africa (MENA) region, as a whole, is burdened with high prevalence rates of physical inactivity [6] reaching 82.3% among Syrian adults [7] and 96.1% in Saudi Arabia [8]. In parallel, the region has the highest comparative prevalence of diabetes (4%–21%) [9]. According to the International Diabetes Federation [10], six out of the world’s top ten countries for highest prevalence of diabetes are from the MENA region.

Lebanon, a small-middle income country of the MENA region, is characterized by high urbanization rate (87%), high literacy rate (89%), and life expectancy approaching 73 years for men and 76 years for women. With westernization and socioeconomic changes, non-communicable diseases have long emerged as the leading cause of morbidity and mortality [11]. Obesity rates have increased from 17·4% in 1997 to 28·2% in 2009 among adults aged 20 years and older [12] and close to one third of the adult population suffer from metabolic syndrome [13]. Yet, national data on the distribution of physical activity and diabetes are limited to a few small-scale surveys and remain deficient. Also, physical activity is influenced by a diverse range of factors, and the influence of these factors on the pattern of physical activity in the general population and among those with diabetes has not been studied. Using data from a recent nationally representative cross-sectional survey, this study intended to address these issues in order to inform public health policies and plans. The study aimed to answer the following questions: firstly, what is the prevalence of physical activity among the total population, and among those with diabetes and risk of developing diabetes; and secondly, what are the factors associated with the level of physical activity performed as stratified by diabetes status?

## Methods

### Study design and participants

The data used in this study were drawn from the national Nutrition and Non-Communicable Disease Risk Factor (NNCD-RF) survey conducted in Lebanon between 2008 and 2009 on a representative sample of Lebanese adults aged 25 and over of both sexes. This was a population-based cross-sectional survey that followed the WHO STEPwise guidelines. The study sample was drawn based on random area probability multi-stage cluster sampling: the strata were the Lebanese Governorates, the clusters were selected at the level of districts, urban and rural, and the housing units constituted the primary sampling units in the different districts. Only one adult from each household was selected, excluding pregnant and lactating women and individuals with mental disabilities. Refusals at the household level did not exceed 10.7%, yielding a sample size of 2195 adults aged 25 years and over. The study sample had enough power to detect an overall diabetes prevalence of 10%, with an alpha of 5% and an error margin of ± 1.5%. The protocol of the NNCD-RF was approved by the Institutional Review Board of the American University of Beirut and all subjects gave informed consent for their participation.

### Measures

#### Physical activity

Physical activity, our main outcome measure, was assessed using the short version of the International Physical Activity Questionnaire (IPAQ) [14]. While the use of devices such as accelerometers and pedometers may provide a more reliable measure of PA, however the IPAQ has been shown to be a reliable and valid tool to obtain comparable estimates of physical activity, based on activity undertaken across multiple domains including leisure, domestic, work and transport [15,16]. The questionnaire asks about three specific levels of activity: walking, moderate- and vigorous-intensity activities, and their frequency (days per week) and duration (minutes per day). For this study, a measure that describes the total volume of physical activity was calculated by weighting each type of activity by its energy requirements defined in METs (METs are multiples of the resting metabolic rate estimated at 3.3 for walking, 4.0 for moderate physical activity and 8.0 for vigorous physical activity) to yield a combined score in MET.minutes (computed by multiplying the MET score by the minutes performed within a week). As regular participation is a key concept in current public health guidelines for physical activity, both the total volume and the number of days/sessions are included in the IPAQ analysis algorithms, thus classifying our subjects into three categories: inactive, moderately active, and HEPA (Health Enhancing Physical Activity) [17]. The moderate level corresponded to meeting physical activity guidelines of 30 minutes of moderate intensity activity 5 days a week or 20 minutes of vigorous activity 3 days a week, or a combination of both. HEPA corresponded to either having vigorous-intensity activity on > 3 days/week while accumulating at least 1500 MET-minutes/week; or >5 days of any combination of walking, moderate-intensity, or vigorous-intensity intensity activities achieving at least 3000 MET-minutes per week. Subjects not meeting either criterion were classified as having low level of physical activity.

#### Adults with diabetes and those at risk of developing diabetes

Respondents were defined as ‘having diabetes’ based on self-reports to the question whether they had ever been diagnosed with diabetes by a health professional, excluding gestational diabetes. Using the American Diabetes Association criteria [18], we also defined individuals at ‘risk of developing diabetes’ as those non-diabetic subjects having one or more of the following six conditions: age over 45 years, overweight (BMI ≥ 25 kg/m^2^), a diagnosis of hypertension, hyperlipidemia, cardiovascular disease and having family history of diabetes in first degree relatives. BMI was based on objective measures of height and weight, using calibrated methods and equipment. Whereas the presence of hyperlipidemia, cardiovascular disease and family history of diabetes were based on self-reports, blood pressure was objectively measured using a standardized mercury sphygmomanometer after participants were seated and rested for 5 minutes. The average of three measurements was considered and hypertension was judged to be present if the participant had a mean value for systolic/diastolic blood pressure ≥ 140/90 mmHg and/or was on medications for hypertension.

#### Other covariates

In addition to the above, other covariates were examined as factors associated with physical activity and these included gender, governorate (Beirut, the capital city vs. others), and education (low, middle, high). Low educational level included those with education less than primary schooling (equivalent to eight years of schooling), middle educational level included those who attended at least secondary or technical schooling (equivalent to 12 years of schooling), while those with a high educational level were holders of a university bachelor’s degree or higher. We also included in our list of covariates ‘having received health professional advice to exercise more’ within the past six months prior to the survey. The number of cases with missing data was not substantial in the independent and dependant variables (n= 43 in either variable), and this was dealt with by employing list-wise deletion.

### Data analysis

Descriptive statistics, expressed as percentages and number of cases, were calculated for the total study population as well as across categories of physical activity levels. Prevalence estimates of physical activity and their 95% confidence intervals (CI) were calculated for diabetic patients and those without diabetes according to their level of risk of developing diabetes. Evidence-based guidelines for physical activity among diabetic subjects indicate the need to be engaged in moderate to vigorous physical activity for at least 3 days a week [19]. Hence, physical activity was further grouped, for the logistic regression analysis, into a binary variable: physically active to include individuals with moderate and high levels of physical activity and the physically inactive. The regression analysis estimated the odds of being physically active among adults in the total sample, and among subjects with and without diabetes, separately. Covariates included in the models were age, gender, governorate, education, car ownership, smoking, BMI, hypertension and hyperlipidemia, heart disease, as well as receiving advice to exercise. Prevalence odds ratios (ORs) and their 95% CIs were estimated. Statistical significance was defined as p-value<0.05, and SPSS 18.0 and STATA 10.0 software packages were used for analyses.

## Results

The mean age of the study sample was 44.7 years (SD±14.9) with a slightly larger proportion of females than males (53.6% vs. 46.4%, respectively) (Table 1). The majority of participants had attained middle or high level education (75.4%), resided outside the capital city of Beirut (89.2%), and owned at least one car (71.5%). Close to 38% were current smokers. Their mean BMI was 27.8 kg/m^2^ (SD=5.4), with around 29% being obese (BMI≥30 Kg/m^2^). Hypertension was the most prevalent health condition (31.8%). This was followed by hyperlipidemia (18.5%) and heart disease (8.0%). Around one third of individuals were advised to exercise in the six months prior to the survey.

**Table 1:** Baseline characteristics of study participants and rates of physical activity, Lebanon, 2009

	**Baseline distribution**		**Rates of physical activity**					
			**Inactive**		**Moderate**		HEPA*	
	**N**	**%**	**n**	**%**	**n**	**%**	**n**	**%**
Total Sample	2195		1026	46.7	682	31.1	487	22.2
**Socio-demographic**								
Age								
25–44	1245	56.7	608	48.8	348	28.0	289	23.2
45–64	664	30.3	271	40.8	239	36.0	154	23.2
≥65	286	13.0	147	51.4	95	33.2	44	15.4
Gender								
Male	1019	46.4	520	51.0	286	28.1	213	20.9
Female	1176	53.6	506	43.0	396	33.7	274	23.3
Governorate								
Beirut	236	10.8	139	58.9	64	27.1	33	14.0
Other	1959	89.2	887	45.3	618	31.5	454	23.2
Education								
Low	540	24.6	237	43.9	184	34.1	119	22.0
Middle	952	43.4	418	43.9	300	31.5	234	24.6
High	703	32.0	371	52.8	198	28.2	134	19.1
Owns a car								
None	547	24.9	215	39.3	197	36.0	135	24.7
1 or more	1648	71.5	811	49.2	485	29.4	352	21.4
**Health-related**								
Smoking								
Never	1217	55.4	557	45.8	401	32.9	259	21.3
Past	150	6.8	78	52.0	41	27.3	31	20.7
Current	828	37.7	391	47.2	240	29.0	197	23.8
BMI								
Normal	719	32.8	336	46.7	216	30.0	167	23.2
Overweight	832	37.9	359	43.1	275	33.1	198	23.8
Obese	638	29.1	328	51.4	190	29.8	120	18.8
Hypertension								
No	1489	67.8	698	46.9	447	30.0	344	23.1
Yes	699	31.8	324	46.4	234	33.5	141	20.2
Hyperlipidemia								
No	1790	81.5	815	45.5	553	30.9	422	23.6
Yes	405	18.5	211	52.1	129	31.9	65	16.0
Heart disease								
No	2019	92.0	926	45.9	628	31.1	465	23.0
Yes	176	8.0	100	56.8	54	30.7	22	12.5
Advice to exercise								
No	1544	70.3	724	46.9	454	29.4	366	23.7
Yes	651	29.7	302	46.4	228	35.0	121	18.6

*HEPA, health enhancing physical activity.

Some totals do not add up to 2195 or 100% due to missing data.

Table 1 also presents physical activity rates according to the various socio-demographic and health characteristics of the study sample (row %s). Close to half of the survey participants reported low or no physical activity (46.7%), 31.1% reported moderate levels, while 22.2% reported HEPA levels. Physical activity, notably HEPA, varied with age being lowest among older adults aged 65 and above. Compared to their counterparts, physical activity was slightly higher among females, those living outside Beirut, and among participants with low-to-middle educational levels. Obese respondents and those suffering from co-morbid conditions including hypertension, hyperlipidemia and heart disease were less likely to be performing HEPA. Respondents receiving medical advice to exercise were more likely to engage in moderate levels of physical activity.

Survey participants with diagnosed diabetes made up 8.5% of the study sample (Table 2). An additional 26.3% and 4.1% of the subjects without diabetes had 3–4 and 5–6 diabetes risk factors, respectively. HEPA prevalence rates decreased consistently with increasing number of diabetes risk factors (p-value<0.001) and was significantly lower among those with diabetes than those without diabetes (9.6% vs. 23.4%, p-value<0.001).

**Table 2:** Rates of physical activity among Lebanese adults diagnosed with diabetes and at risk for developing diabetes, 2009

	**Baseline population**		**Rates of physical activity**									
			**Inactive**			**Moderate**			**HEPA**			
	**N**	**%**	**n**	**%**	**95% CI**	**n**	**%**	**95% CI**	**n**	**%**	**95% CI**	**P-value**
Diabetes	187	8.5	104	55.6	48.0–63.0	65	34.8	28.0–42.0	18	9.6	5.8–14.8	<0.001
No Diabetes	2008	91.5	922	45.9	43.7–48.1	617	30.7	28.7–32.8	469	23.4	21.5–25.3	
0 diabetes risk factors*	229	10.4	109	47.6	40.9–54.3	54	23.6	18.2–29.6	66	28.8	23.0–35.1	<0.001
1 to 2 diabetes risk factors	1111	50.6	513	46.2	43.2–49.1	337	30.3	27.6–33.1	261	23.5	21.0–26.1	
3 to 4 diabetes risk factors	577	26.3	256	44.4	40.3–48.5	197	34.1	30.3–38.2	124	21.5	18.2–25.1	
5 to 6 diabetes risk factors	91	4.1	44	48.4	37.7–59.1	29	31.9	22.5–42.5	18	19.8	12.2–29.5	
Total	2195	100	1026	46.7	44.6–48.9	682	31.1	29.1–33.0	487	22.1	20.5–24.0	

*Diabetes risk factors, based on the ADA, included: age ≥ 45 years, BMI≥25 kg/m^2^, a diagnosis of hypertension, hyperlipidemia, and cardiovascular disease and family history of diabetes.

Results of the multivariable logistic regression model for the total sample and stratified by diabetes status are shown in Table 3. In the total sample, physical activity was significantly higher among the middle-age group (45–64 years) (OR=1.46, 95% CI 1.17–1.81), females (OR=1.30, 95% CI 1.09–1.55), and among respondents residing in governorates outside the capital city of Beirut (OR=1.61, 95% CI 1.21–2.62). In contrast, participants with high levels of education (OR=0.75, 95% CI 0.57–0.98) and those who owned at least one car (OR=0.71, 95% CI 0.57–0.88) were significantly less likely to be physically active. Whilst hypertension did not associate with physical activity, obesity (OR=0.78, 95% CI 0.62–0.99) and pre-existing health conditions significantly decreased the odds of physical activity (OR=0.66, 95% CI 0.51–0.85 for hyperlipidemia; OR=0.61, 95% CI 0.43–0.87 for heart disease). A significant association was also noted for receiving health professional advice to engage in physical activity (OR=1.25, 95% CI 1.01–1.54).

**Table 3:** Correlates of physical activity in the total sample and stratified by diabetes status among Lebanese adults: results of the logistic regression, 2009

	**Total sample**		**Subjects without diabetes**		**Subjects with diabetes**	
	**n=2195**		**n=2008**		**n=187**	
	**Adjusted OR**	**95% CI**	**Adjusted OR**	**95% CI**	**Adjusted OR**	**95% CI**
Age						
25–44	1.00		1.00		1.00	
45–64	1.46	1.17–1.81	1.46	1.64–1.84	1.51	0.53–4.34
≥65	0.98	0.71–1.35	0.92	0.64–1.32	1.42	0.45–4.46
Gender						
Male	1.00		1.00		1.00	
Female	1.30	1.09–1.55	1.38	1.14–1.66	0.64	0.31–1.33
Governorate						
Beirut	1.00		1.00		1.00	
Other	1.61	1.21–2.13	1.63	1.21–2.18	1.57	0.55–4.47
Education						
Low	1.00		1.00		1.00	
Middle	1.02	0.80–1.29	0.95	0.74–1.22	1.36	0.64–2.89
High	0.75	0.57–0.98	0.72	0.54–0.96	0.47	0.17–1.31
Owns a car						
No	1.00		1.00			
One or more	0.71	0.57–0.88	0.69	0.55–0.86	0.96	0.45–2.06
Smoking						
Never	1.00		1.00		1.00	
Past	0.83	0.58–1.20	0.85	0.70–1.03	0.83	0.31–2.25
Current	0.87	0.72–1.06	0.88	0.59–1.32	1.02	0.48–2.11
BMI						
Normal	1.00		1.00		1.00	
Overweight	1.16	0.94–1.43	1.16	0.93–1.44	1.08	0.40–2.87
Obese	0.78	0.62–0.99	0.81	0.64–1.04	0.50	0.19–1.31
Hypertension						
No	1.00		1.00		1.00	
Yes	0.96	0.74–1.25	1.17	0.93–1.47	0.85	0.41–1.78
Hyperlipidemia						
No	1.00		1.00		1.00	
Yes	0.66	0.51–0.85	0.69	0.52–0.93	0.67	0.34–1.36
Heart Disease						
No	1.00		1.00		1.00	
Yes	0.61	0.43–0.87	0.81	0.54–1.22	0.29	0.13–0.70
Advice to Exercise						
No	1.00		1.00		1.00	
Yes	1.25	1.01–1.54	1.17	0.93–1.46	3.68	1.55–8.74

Findings of the regression analysis varied according to diabetes status. Owing to the relatively large proportion of subjects without diabetes in our study sample (91%), the direction and strength of the associations among the non-diabetic subjects were, overall, similar to those estimated for the total sample. Among diabetic subjects, we note that the results were, in the majority, not significant and with wide confidence intervals. However, the association of physical activity with heart disease (OR=0.29, 95% CI 0.13–0.70) and with receiving professional advice to exercise were further enhanced among subjects with diabetes (OR=3.68, 95% CI 1.55–8.74), compared to those without diabetes.

## Discussion

Findings of this study indicate that close to one third (31%) of Lebanese adults, 25 years of age and older, participate in moderate levels of physical activity and only 22% engage in health enhancing energy expenditure levels. These results are lower than those reported in the International Prevalence Study conducted in 20 countries worldwide using the same instrument as ours (median 33% and 40% for moderate and vigorous activity, respectively) [20]. Compared to their counterparts, physical activity was lower in subgroups with urban characteristics and lifestyle, namely those with high levels of education, who owned at least one car, and among those who resided in Beirut, the capital city. Given the clear evidence in the literature of the health benefits of physical activity in the prevention and management of diabetes, it is disturbing to note that HEPA rates decreased consistently with increasing number of diabetes risk factors and were only 10% among subjects known to have diabetes.

During the last few years, different subgroups of the Lebanese adult population have been studied with regard to physical activity with varying results. For example, Sibai and colleagues found approximately 16% of HEPA levels in a sample of 499 adult population attending health centers in Lebanon [13]. Tohme and colleagues [21] estimated the prevalence rate of daily exercise for 30 minutes in a nationally representative sample of 2,125 subjects at 20.4%. A cross sectional study conducted among 346 adults 18 years and over selected from large stores across all governorates yielded a much higher estimate (55%) [22]. Findings from the WHO STEPwise surveys in countries of the MENA region also show a wide range of prevalence estimates of physical activity, varying between 13.2% and 78.4% [23]. The outcomes of these studies are difficult to compare with findings from the present study owing to differences in sampling design, sample characteristics, as well as the tool, operational definition and the scoring method employed to measure physical activity.

We found inverse associations between physical activity and various indicators of socio-economic status (SES), as illustrated by education (OR=0.75), car ownership (OR=0.71), and residence in Beirut, the highly urbanized metropolitan capital city of the country (OR=0.62). Reviews of SES correlates with physical activity show mostly positive associations; this is particularly evident for education [24,25]. Yet, this evidence has largely been noted for leisure-time physical activity with diverse and often crude measures being employed [6,24]. In comparison, our assessment of physical activity was based on the IPAQ that includes energy expenditure from several domains, and captures expenditure from lifestyle activities integrated in daily routines of work, household chores and transportation, additional to leisure time activities. Thus, higher education, car ownership and residence inside the capital city can mean more sedentary jobs, reduced walking and less physically active lifestyles. Support for our results is found in studies from sub-Saharan Africa where urban dwellers had a significantly lower physical activity energy expenditure than rural dwellers (44.2 ± 21.0 vs. 59.6 ± 23.7 kJ) [26], and in selected developing countries of the Asian-Pacific and the MENA regions [14,27]. Similarly, cross-country comparisons at the ecological level show that physical activity is more common in low income countries than in high income countries [6,28].

Consistent with findings from the literature [3], the presence of obesity and co-morbidities associated inversely with physical activity among our study participants [3]. Also, HEPA levels declined consistently as the number of diabetes risk factors increased, and was lowest among people with diabetes. Given the benefits of regular moderate physical exercise as a form of intervention management for improving HbA1c levels and cardiovascular risk factors in diabetic patients, it is concerning that the majority of Lebanese adults with diabetes remain either sedentary or insufficiently active (55.6%) and only 9.6% participate in health enhancing activities. Our results are similar to those found among 406 consecutive patients attending the Diabetes clinic in Dundee, UK (9%) [29], yet much lower than findings from other patient groups in the National Health and Nutrition Examination Survey (28.2%) and in the Medical Expenditure Panel Survey (39%) in the US [30,31].

Patients receiving advice from a health professional to alter their lifestyle and exercise were found to be significantly more likely to engage in physical activity than non-diabetic subjects receiving the same advice, net of the effect of other covariates (OR=3.68 and OR=1.17, respectively). This finding merits further consideration. Post hoc analysis of our data shows a positive gradient between receiving advice and the presence of diabetes risk factors (p-value for trend <0.01) with health professionals advising most patients with or at highest risk for diabetes (76.5% and 71.4%, respectively) and least those without diabetes (13.1%). While this implies the recognition by health professionals of the importance of physical activity among diabetes patients on one end of the spectrum, it also suggests a missed opportunity for primary disease prevention in the clinic for the majority non-diabetic population, on the other end. Given the crucial role of physical activity in the prevention and management of diabetes, these results have important implications to health care providers and policy makers with regard to diabetes control guidelines and protocols. Whereas studies are needed to examine contextual factors that influence behavioral change of patients and non-patients in Lebanon, it is the time to start targeting interventions that promote physical activity in the general as a life-style to be adopted. The challenge remains to alert physicians towards primary disease prevention services for the non-diabetic majority and to re-direct policy attention to diabetes control from the high-risk emphasis towards the population-based public health approach.

The findings of this study are considered in light of the following limitations. Because of the cross-sectional nature of the study, the temporality of certain associations cannot be established with confidence. Also, the study lacked sufficient power to detect significant correlates of physical activity among subjects with diabetes (n=187). Whereas BMI and hypertension were objectively measured using calibrated equipment and standardized techniques, data on diabetes status are self-reported, introducing possible misclassification bias. However, the amount of bias is minimal as post-hoc analysis of our data showed that those who reported having diabetes were mostly on medication and/or were following a diet (84%). Also, diabetes is an overt disease, and so diabetic individuals are likely to be referred to a physician for diagnosis and follow-up. Furthermore, studies from Lebanon and elsewhere show substantial agreement between self-report and medical record diagnosis for several medical conditions including diabetes (kappa range 0.71–0.78) and heart disease (kappa range 0.83–0.87) [32-34]. Although the IPAQ’s validity has originally been assessed for individuals aged up to 69 years of age, nonetheless, studies in the developed world and in the region have used and validated it on samples aged over 70 years [35,36].

In spite of the above caveats, this is the first study that we are aware of to assess the prevalence of physical activity and its correlates with a focus on diabetes and diabetes risk factors in a large national sample in Lebanon and the MENA region. Estimates of physical activity from national surveys such as the current one can provide valuable information to guide national policies and serve as a benchmark for monitoring and evaluation thereafter. Furthermore, our assessment of physical activity based on the IPAQ captures more contributions towards total physical activity levels and does not ignore routine activities undertaken by housewives, for example, who constitute a good percent of the Lebanese female population (67.5%). The ability to quantify benefit from several domains including routine daily pursuits, whether at work or home, may be more applicable to health promotion initiatives in low-income countries such as Lebanon where access to leisure-time activity is hampered by the cost of private club memberships and the lack of public and age-friendly outdoor spaces to exercise.

## Conclusions

In conclusion, population-based approaches for control and prevention of diabetes by physical activity should be innovative while addressing the patient's concepts and community perspectives of health and illness and understanding the complex interaction between barriers and facilitators across the different levels of the individual, family, community and society. Structural factors may limit our population’s engagement of physical activity [37], hence the need for governmental policies that are appropriate to population’s context. Yet the statement made by the National Board of Health and Welfare in Sweden as early as the 1970s to “take every opportunity to increase life style activities” remains the most forceful and affordable message to be adopted by policymakers at the population-level in developing countries.

## Abbreviations

BMI: Body mass index; HEPA: Health enhancing physical activity; IPAQ: International physical activity questionnaire; MENA: Middle east and North Africa; MET: Metabolic equivalent; NNCD-RF: Nutritional and Non-communicable disease risk factor.

## Competing interests

The authors declare that they have no competing interests.

## Authors’ contributions

AMS contributed towards study design, study logistics, data collection, data analysis, and interpretation and manuscript drafting. RT contributed toward study logistics, data collection, analysis and interpretation. SA contributed to conception of the hypothesis, the analysis and write-up of the paper. NH contributed to study logistics, data collection and data interpretation. CC contributed to the conception of the hypothesis, analysis and write-up. All authors provided critical insight, and revisions to the manuscript. All authors read and approved the final version of the manuscript submitted for publication.

## Pre-publication history

The pre-publication history for this paper can be accessed here:

http://www.biomedcentral.com/1471-2458/13/1002/prepub

## References

[B1] Morris JN Heady JA Raffle PA Roberts CG Parks JW Coronary heart-disease and physical activity of work Lancet 1953 13 1053 1057 13110049 10.1016/s0140-6736(53)90665-5

[B2] Lee IM Shiroma EJ Lobelo F Puska P Blair SN Katzamarzyk PT Effect of physical inactivity on major non-communicable diseases worldwide: an analysis of burden of disease and life expectancy Lancet 2012 13 219 229 10.1016/S0140-6736(12)61031-9 22818936 PMC3645500

[B3] Luke A Dugas LR Durazo-Arvizu RA Cao G Cooper RA Assessing physical activity and its relationship to cardiovascular risk factors: NHANES 2003-2006 BMC Public Health 2011 13 387 10.1186/1471-2458-11-387 21612597 PMC3123595

[B4] Kohl HW 3rd Craig CL Lambert EV The pandemic of physical inactivity: global action for public health Lancet 2012 13 294 305 10.1016/S0140-6736(12)60898-8 22818941

[B5] Centers for Disease Control and Prevention Surgeon General’s Report on Physical Activity and Health 1996 Springfield, VA: National Technical Information Service

[B6] Hallal PC Anderson LB Bull FC Global physical activity levels: surveillance progress, pitfalls, and prospects Lancet 2012 13 247 257 10.1016/S0140-6736(12)60646-1 22818937

[B7] Al Ali R Rastam S Fouad MF Mzayek F Maziak W Modifiable cardiovascular risk factors among adults in Aleppo, Syria Int J Public Health 2011 13 653 662 10.1007/s00038-011-0278-0 21814848

[B8] Mabry RM Reeves MM Eakin EG Owen N Evidence of physical activity participation among men and women in the countries of the gulf cooperation council: a review Obes Rev 2010 13 6 457 464 19793376 10.1111/j.1467-789X.2009.00655.x

[B9] Badran M Laher I Type II diabetes mellitus in Arabic-speaking countries Int J Endo 2012 doi:10.1155/2012/902873 10.1155/2012/902873 PMC3407623 22851968

[B10] International Diabetes Foundation Rates of diabetes in the Middle East to double in next 20 years 2011 Accessed on August 1st 2012: http://www.idf.org/sites/default/files/attachments/MENA-Press-Release-WDD.pdf

[B11] Sibai AM Fletcher A Hills M Campbell O Non-communicable disease mortality rates using the verbal autopsy in a cohort of middle-aged and older populations in Beirut during wartime, 1983–93 J Epidemiol Community Health 2001 13 271 276 10.1136/jech.55.4.271 11238583 PMC1731870

[B12] Nasreddine L Naja F Chamieh MC Adra N Sibai AM Hwalla N Trends in overweight and obesity in Lebanon: evidence from two national cross-sectional surveys (1997 and 2009) BMC Public Health 2012 13 1 798 doi:10.1186/1471-2458-12-798 10.1186/1471-2458-12-798 22984791 PMC3527186

[B13] Sibai AM Obeid O Batal M Adra N El Khoury D Hwalla N Prevalence and correlates of metabolic syndrome in an adult Lebanese population CVD Prev Control 2008 13 83 90 10.1016/j.precon.2007.06.002

[B14] Bauman A Cuevas F Omar Z Cross-national comparison of socioeconomic differences in the prevalence of leisure-time and occupational physical activity, and active commuting in six Asia-Pacific countries J Epidemiol Community Health 2011 13 35 4312 10.1136/jech.2008.086710 20943821

[B15] Hagströmer M Oja P Sjöström M The international physical activity questionnaire (IPAQ): a study of concurrent and construct validity Public Health Nutr 2005 13 755 762 10.1079/phn2005898 16925881

[B16] Papathanasiou G Georgoudis G Papandreou M Reliability measures of the short international physical activity questionnaire (IPAQ) in Greek young adults Hellenic J Cardiol 2009 13 283 294 19622498

[B17] Guidelines for data processing and analysis of the international physical activity questionnaire (IPAQ)– short and long forms 2005 Accessed at http://www.ipaq.ki.se/scoring.pdf

[B18] American Diabetes Association Screening for type 2 diabetes Diabetes Care 2003 13 s21 s24 12502615 10.2337/diacare.26.2007.s21

[B19] American Diabetes Association and National Institute of Diabetes, Digestive & Kidney Diseases The prevention or delay of type 2 diabetes Diabetes Care 2002 13 742 749 11919135 10.2337/diacare.25.4.742

[B20] Bauman A Bull F Chey T The international prevalence study on physical activity: results from 20 countries Int J Behav Nutr Phy 2009 13 21 10.1186/1479-5868-6-21 PMC2674408 19335883

[B21] Tohme RA Jurjus AR Estephan A The prevalence of hypertension and its association with other cardiovascular disease risk factors in a representative sample of the Lebanese population J Hum Hypertens 2005 13 861 868 10.1038/sj.jhh.1001909 16034449

[B22] Al-Tannir M Kobrosly S Itani T El-Rajab M Tannir S Prevalence of physical activity among Lebanese adults: a cross-sectional study J Phys Act Health 2009 13 315 320 19564659 10.1123/jpah.6.3.315

[B23] Sibai AM Nasreddine L Mokdad HA Adra N Tabet M Hwalla N Nutrition transition and cardiovascular disease risk factors in middle east and north Africa countries: reviewing the evidence Ann Nutr Metab 2010 13 193 203 10.1159/000321527 21088386

[B24] Trost SG Owen N Bauman AE Sallis JF Brown W Correlates of adults’ participation in physical activity: review and update Med Sci Sports Exerc 2002 13 1996 2001 10.1097/00005768-200212000-00020 12471307

[B25] Gidlow C Johnston LH Crone D Ellis N James D A systematic review of the relationship between socio-economic position and physical activity Health Educ J 2006 13 338 367 10.1177/0017896906069378

[B26] Assah FK Ekelund U Brage S Mbanya JC Wareham NJ Urbanization, physical activity, and metabolic health in Sub-Saharan Africa Diabetes Care 2011 13 491 496 10.2337/dc10-0990 21270205 PMC3024374

[B27] Merom D Sinnreich R Aboudi V Kark JD Nassar H Lifestyle physical activity among urban Palestinians and Israelis: a cross-sectional comparison in the Palestinian-Israeli Jerusalem risk factor study BMC Public Health 2012 13 90 10.1186/1471-2458-12-90 22289260 PMC3311574

[B28] Dumith SC Hallal PC Reis RS Kohl HW 3rd Worldwide prevalence of physical inactivity and its association with human development index in 76 countries Prev Med 2011 13 24 28 10.1016/j.ypmed.2011.02.017 21371494

[B29] Thomas N Alder E Leese GP Barriers to physical activity in patients with diabetes Postgrad Med J 2004 13 287 291 10.1136/pgmj.2003.010553 15138320 PMC1742997

[B30] Resnick HE Foster GL Bardsley J Ratner RE Achievement of American diabetes association clinical practice recommendations among U.S. Adults with diabetes, 1999–2002: the national health and nutrition examination survey Diabetes Care 2006 13 531 537 16505501 10.2337/diacare.29.03.06.dc05-1254

[B31] Morrato EH Hill JO Wyatt HR Ghushchyan V Sullivan PW Physical activity in U.S. adults with diabetes and at risk for developing diabetes, 2003 Diabetes Care 2007 13 203 209 10.2337/dc06-1128 17259482

[B32] Halabi S Zurayk H Awaida R Darwich M Saab B Reliability and validity of self and proxy reporting of morbidity data: a case study from Beirut, Lebanon Int J Epidemiol 1992 13 607 12 10.1093/ije/21.3.607 1386064

[B33] Okura Y Urban LH Mahoney DW Jacobsen SJ Rodeheffer RJ Agreement between self-report questionnaires and medical record data was substantial for diabetes, hypertension, myocardial infarction and stroke but not for heart failure J Clin Epidemiol 2004 13 1096 1103 10.1016/j.jclinepi.2004.04.005 15528061

[B34] Tisnado DM Adams JL Liu H What is the concordance between the medical record and patient self-report as data sources for ambulatory care? Med Care 2006 13 132 140 10.1097/01.mlr.0000196952.15921.bf 16434912

[B35] Ainsworth BE Macera CA Jones DA Comparison of the 2001 BRFSS and the IPAQ physical activity questionnaires Med Sci Sports Exer 2006 13 1584 1592 10.1249/01.mss.0000229457.73333.9a 16960519

[B36] Al-Hazzaa HM Heath-enhancing physical activity among Saudi adults using the international physical activity questionnaire (IPAQ) Public Health Nutr 2007 13 59 64 17212844 10.1017/S1368980007184299

[B37] Sibai AM Hwalla N Adra N Rahal M Prevalence and covariates of obesity in Lebanon: findings from the first epidemiological study Obes Res 2003 13 1353 1361 10.1038/oby.2003.183 14627756

